# Electrochemical properties of an activated carbon xerogel monolith from resorcinol–formaldehyde for supercapacitor electrode applications†

**DOI:** 10.1039/d1ra06462b

**Published:** 2021-10-08

**Authors:** Minhu Huang, Seung Joon Yoo, Jae-Suk Lee, Tae-Ho Yoon

**Affiliations:** School of Materials Science and Engineering, Gwangju Institute of Sci. and Eng. (GIST) 123 Cheomdangwagi-ro, Buk-gu Gwangju 61005 South Korea thyoon@gist.ac.kr +82-62-715-2304 +82-62-715-2307

## Abstract

Activated carbon xerogel monoliths were prepared from resorcinol and formaldehyde *via* a catalyst-free and template-free hydrothermal polycondensation reaction, followed by pyrolysis and activation. The ratio of resorcinol (R) to distilled water (W) was varied to afford an interconnected pore structure with controlled pore size, while the pyrolysis temperature was optimized to give high specific surface area. Activation was carried out at 700 °C after soaking the samples in 6 M KOH aqueous solution. The same process, called “heat treatment”, was also carried out without soaking in KOH for comparison. The weight loss upon pyrolysis, activation and heat treatment and the weight gain *via* KOH soaking were measured. Field emission scanning electron microscopy (FE-SEM), X-ray photoelectron spectroscopy (XPS), Raman spectroscopy, X-ray diffraction (XRD), thermogravimetric analysis (TGA) and an N_2_ sorption instrument were utilized for characterization. Additionally, electrochemical properties were evaluated using cyclic voltammetry (CV), galvanostatic charge–discharge (GCD) and electrochemical impedance spectroscopy (EIS) with a 3-electrode system, while a 2-electrode system was also employed for selected samples. The highest specific capacitance of 323 F g^−1^*via* GCD at 1 A g^−1^ was obtained at the R/W ratio of 45 and with 500 °C pyrolysis. In addition, this sample also exhibited 89.4% retention at 20 A g^−1^ in the current density variation and 100% retention in 5000 cycling tests.

## Abstract 2

A monolithic carbon xerogel electrode for supercapacitors was prepared from resorcinol–formaldehyde, providing a specific capacitance of 323 F g^−1^*via* GCD at 1 A g^−1^ and 100% retention upon 5000 cycling tests.

## Graphical abstract

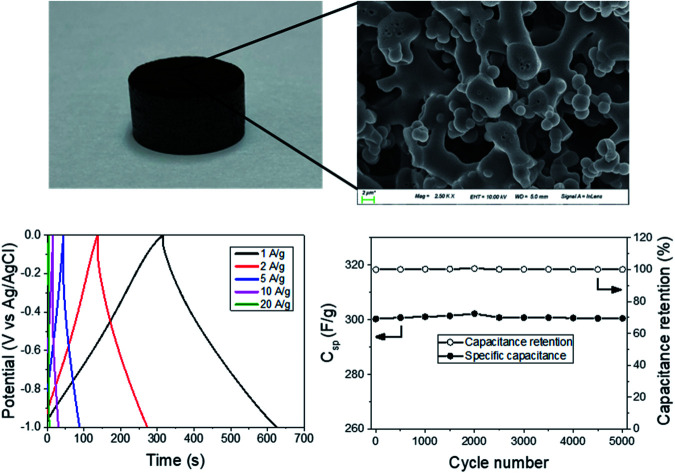

## Introduction

Supercapacitors are excellent energy storage devices for portable electronics, communication devices, electrical vehicles, renewable energy systems and other devices due to their faster charge–discharge rate, higher power density and longer life cycle than batteries.^1,2^ However, their lower energy density is a major drawback.^3^ This has led to a great deal of research, indicating that the energy density can be enhanced by increasing the number of ions adsorbed to the electrode for electrostatic double layer capacitors (EDLCs) or charge-transfer between the electrode and the electrolyte for electrochemical pseudocapacitors. What is imperative for the electrode is to have a large surface area, which in turn requires the electrode to have more micro-pores than meso- or macro-pores. However, micro-pores are known to give rise to the high flow resistance of the electrolyte, thereby deteriorating the energy density. Meso- or macro-pores, on the other hand, do not contribute much to the surface area, but provide a large path for the electrolyte, thus, easier flow of the electrolyte. Therefore, a suitable combination of these pores, so called “hierarchical pore structure”,^4^ is necessary to not only create a high surface area but also facilitate easy flow of the electrolyte. Of course, it would be even better if the pores were interconnected to provide easier flow of the electrolyte. In addition to this, other key factors that need to be considered in selecting electrode materials include good electrical conductivity, high temperature and chemical stability, good environmental friendliness and low cost.^5^ In light of this, the activated carbon is an excellent electrode candidate for EDLCs and pseudocapacitors. In the latter case, however, activated carbons need to be used as a porous template^6^ because of their poor pseudocapacitive property.^7^

Activated carbons used to be produced from wood and fossil raw materials, but their depletion and/or high price led to an effort to find alternatives. One such alternative is biomass or bio-waste because they are abundant, inexpensive, renewable and sustainable.^8^ A number of these have been studied to prepare activated carbons for the electrodes used in supercapacitors.^9^ However, activated carbons from these materials are powdery, in general, and thus, require a binder to fabricate the electrodes. Unfortunately, the binder not only increases the electrode weight and its production cost, but also deteriorates electrochemical performance.^10^ Therefore, the need to prepare the electrode without the use of binders led to the preparation of carbon monolith^11–13^ which was initially introduced for the powdery activated carbons made from polymers.^14^

In fact, such polymers have been studied for much longer than the biomasses or bio-wastes, owing to the high quality of resulting activated carbons. However, they did not gain high popularity until Pekala reported the successful preparation of carbon monolith *via* a simple hydrothermal polycondensation reaction of resorcinol and formaldehyde.^14^ Of course, phenol^15^ and phlorogluconol^16^ were also studied, but their water-insolubility and/or higher price made them less popular than resorcinol.^17^ Unfortunately, the reaction is rather complicated, requiring a catalyst to promote a fast reaction, template to form micro- and/or meso-pores, and/or a special drying process to avoid pore collapsing. In fact, the reaction by Pekala employed a catalyst and supercritical drying^14^ without a template to obtain a low surface area, which resulted in allow specific capacitance of 95 F g^−1^. Subsequently, a number of studies followed in an attempt to enhance the surface area, such as the adaption of templates.^17^ However, Bauman^18^ prepared a carbon monolith with high surface area (3125 m^2^ g^−1^) without using a template. This was possible because of the interconnected pore structure obtained *via* spinodal decomposition and CO_2_ activation. Unfortunately, no capacitance data was reported. Of course, other polymers such as divinylbenzene,^19^ polyvinylalcohol^20^ and polyacrylonitrile,^21^ as well as the mesophase pitch,^22^ were also studied for the preparation of carbon monolith, but they required the use of a template.

This led to a great deal of research on polymer-based activated carbons during the past decades.^23^ However, carbon monoliths prepared without the use of a catalyst, template and special drying process had not been reported until we prepared a carbon xerogel monolith (CM) using a process that excludes all three of these.^24^ The CM was prepared from resorcinol and formaldehyde *via* the hydrothermal polycondensation reaction. The R/F ratio was varied to afford spinodal decomposition, which resulted in an interconnected pore structure. A high specific surface area of 3400 m^2^ g^−1^ was obtained *via* pyrolysis at 900 °C, followed by CO_2_ activation at 1000 °C, but its electrochemical properties have not been evaluated for supercapacitors. In this study, therefore, the RF-based CM was prepared according to the previous study^24^ but with some modifications such as the variation of R/W ratio and KOH activation. Then, electrochemical properties were evaluated with a three-electrode system *via* cyclic voltammetry (CV), galvanic charge–discharge (GCD) and electrochemical impedance spectroscopy (EIS). A two-electrode system was also studied, but only for selected samples. The samples were also characterized using SEM, TGA, XRD, Raman, XPS and N_2_ sorption instrument.

## Experimental

### Materials

For the synthesis of RF xerogel monoliths, resorcinol (R, Sigma-Aldrich, >99%) and formaldehyde (F, Alfa Aesar, 36.5–38.0 wt% in H_2_O, containing 10–15% of methanol as a stabilizer) were utilized along with deionized water (W, resistivity of 18 MΩ cm *via* Milli-Q Advantage System). A cellulose acetate filter (Advantech, USA) and CR2032 coin cells were utilized for full cell fabrication in the electrochemical study. Potassium hydroxide (KOH, Sigma-Aldrich, 90%) and hydrochloric acid (HCl, Sigma-Aldrich, 37%) were purchased and used without further purification.

### Preparation of activated carbon xerogel monolith (AC)

The preparation of RF xerogel monoliths from resorcinol (R), formaldehyde (F) and deionized water (W) followed the method reported in the literature^24^ with slight modifications, such as variation of the R/W ratio, instead of the R/F ratio, and KOH activation rather than CO_2_ activation. The reason for the former was to control the pore structure to provide a hierarchical pore structure with controlled pore size, while the latter was to obtain even activation through the sample thickness. Briefly, R, F and W were charged into a 50 ml glass vial at the R/F ratio of 0.5 and the R/W ratios of 45, 50, 55 or 60 (%). After complete dissolution, the mixture was poured into a Teflon mould with holes (16 mm in diameter, 40 mm in depth). Al plates (5 mm thick) were then placed on the top and bottom of the mould and bolted to prevent the pressure loss during curing. The mould was placed in an air convection oven at 100 °C for 6 h for curing. After removing the Al plates, the RF-samples in the mould were dried for 36 h at 60 °C, followed by an additional 12 h at 100 °C. The conditions were optimized in advance to avoid crack generation during the drying process.

Next, the column shape RF xerogel monoliths were subjected to pyrolysis in a tube furnace (MSTF-1100, Myungsung Eng., Korea) at 500, 600, 700 or 800 °C for 4 h at a heating rate of 5 °C min^−1^ under the N_2_ flow of 200 sccm. In fact, 400 °C was also attempted, but the resultant CMs were too fragile to make an electrode and excluded from further study. The CMs were cut into 1 mm thick disc samples for the characterization as well as measurement of electrochemical property. The samples were immersed in 6 M KOH aqueous solution overnight under reduced pressure. After drying at RT and then 90 °C for overnight, the samples were activated in a tube furnace at 700 °C for 1 h at a heating rate of 5 °C min^−1^ under the N_2_ flow of 200 sccm. The activated carbon xerogel monoliths (ACs) obtained were then immersed in 1 M HCl solution overnight to remove the residual K and washed copiously. The heat treatment (HT) of the CMs was also carried out at 700 °C for 1 h without KOH, in order to produce heat-treated carbon xerogel monoliths (HCs). The samples were named xx-yy-zzz, where xx, yy and zzz denote the sample type (CM, HC, AC), R/W ratio (45, 50, 55, 60) and pyrolysis temperature (500, 600, 700, 800 °C), respectively.

### Characterization of activated carbon xerogel monolith

The ACs were analysed by FE-SEM (Jeol, JSM-7500F, Hitachi S-4700) at 10 keV with carbon coating, Raman spectroscopy (UniRam-5000, UniThink, Korea, JASCO NRS-5100) equipped with 532 nm laser, and XRD (Empyrean, X'Pert PRO multi purpose) with a Cu (1.8 kW) target. In addition, XPS (Thermo Scientific, K-Alpha^+^, USA) with a monochromatized Al Kα source (1486.6 eV)was also utilized to provide a survey scan as well as narrow scans of C 1s and O 1s peaks. The C 1s peak was subjected to deconvolution for the identification of oxygen functional moieties. It was also studied with thermogravimetric analyzer (PerkinElmer TGA-4000) under N_2_ flow of 50 cm^3^ min^−1^. Pore characteristics of the ACs and CMs were studied with an absorption instrument (ASAP-2010, Micromeritics, USA) using N_2_ at −196 °C. The specific surface area (SSA) and pore size distribution were calculated by the Brunauer–Emmett–Teller (BET) and Barrett–Joyner–Halenda (BJH) methods, respectively, while the mean pore diameter was obtained from 4V/SSA. The total pore volume was calculated from the total single point adsorption of pores with less than 300 nm radius at *P*/*P*_o_ = 0.99, while micro-pore volumes were obtained *via* the *t*-plot theory. All samples were studied with TGA (PerkinElmer TGA-4000, USA) at 10 °C min^−1^ under N_2_ flow up to 700 °C.

### Electrochemical property measurement

The electrochemical performance of ACs was evaluated with 6 M KOH electrolyte at RT using the Versa STAT 3 Instrument (Princeton Applied Research, USA) *via* a three-electrode system. Disk shape ACs (approximately 10 mm in diameter, 10 mg in weight) were prepared by sanding and then utilized as a working electrode. A platinum gauze (1 × 1 cm) and Ag/AgCl (saturated KCl) were used as counter and reference electrodes, respectively. The CV was carried out in the −1 to 0 V range at a scan rate of 2–100 mV s^−1^, while the GCD was performed at the current density of 1–20 A g^−1^. The electrochemical impedance spectroscopy (EIS) was also recorded in the 0.01–100 kHz range with an amplitude of 10 mV. The specific capacitance (*C*_sp_) was calculated from the GCD study based on the formula of *C*_sp_= (*I* × *t*)/(Δ*V* × *m*),^25^ where *I* is the charge–discharge current, *t* is the time of discharge, Δ*V* is the discharge voltage difference between the discharge time and *m* is the mass of the active material.

The samples with the highest *C*_sp_ from the three-electrode system were subjected to the study *via* the two-electrode system. The samples were prepared using a CR2032 coin cell, cellulose acetate filter (Advantech, USA) and two AC discs (approximately 10 mm in diameter and 10 mg in weight). The same conditions as those for the three-electrode system were utilized for the CV, GCD and EIS with the two-electrode system, except for the 0–1 V range in the CV. Results from the GCD were used for the calculation of *C*_sp_, energy and power density, as reported previously.^25,26^ Cyclic stability with the two-electrode system was also evaluated up to 5000 cycles of charging–discharging process at the current density of 1 A g^−1^.

## Results and discussion

Column shape xerogel monoliths were successfully prepared from resorcinol–formaldehyde (RF) *via* the catalyst-free and surfactant-free hydrothermal polycondensation reaction^24^ with R/W ratio variation. Pyrolysis at 500 °C for 4 h resulted in carbon xerogel monoliths (CMs) with a weight loss of ∼32%, regardless of the R/W ratio (Table S1†), which is likely due to the same R/F ratio. However, the weight loss increased with higher pyrolysis temperature (PYT), exhibiting ∼41, ∼46 and ∼48% loss at 600, 700 and 800 °C, respectively, and was not affected by the R/W ratio (Table S1†). This is because degree of carbonization increases with higher PYT, while degree of incomplete carbonization decreases with higher PYT. A shrinkage in diameter was also noted. It increased with higher PYT, but no appreciable change was observed as the R/W ratio changed (Table S2†), possibly due to the fact that the R/F ratio remained the same at 0.5.

For the KOH activation, the CMs were immersed in an aqueous solution of 6 M KOH, which resulted in a decreased weight gain of ∼94, ∼43, ∼22 and ∼19% for R/W = 45, 50, 55 and 60, respectively (Table S3†). This decrease can be explained by the decreased pore size and interconnectivity with higher R/W ratio, which is supported by the SEM micrographs (to be discussed in the later section). However, the weight gains were similar to each other, irrespective of the PYT. This is again attributed to no change in the pore size and interconnectivity with PYT change, as shown in the SEM micrographs (to be discussed later). It is interesting to note that the KOH solution became light brown with the immersion of CM-45-500 and turned lighter in colour with samples prepared at higher PYT, while showing no colour with CM-45-800. This is believed to be caused by the dissolution of partially cured materials, which increased with lower PYT, and can be related to the decreased weight loss with lower PYT (Table S1†), or increased incomplete carbonization with lower PYT.

The variation of the R/W ratio had no effect on the weight loss during pyrolysis. But upon activation at 700 °C for 1 h, the weight loss decreased with higher R/W ratio (Table S4†). This is believed to be caused by a lower degree of activation that occurred due to the lower KOH gain as the R/W ratio increased (Table S3†), being similar to that was reported earlier.^27^ Interestingly, the weight loss also decreased with higher PYT (Table S4†), which was contrary to the trend expected from the pyrolysis. This cannot be related to the KOH gain since similar values were obtained irrespective of the PYT, which would mean that similar weight losses should be obtained upon activation. A possible explanation is that the behaviour arises from the incomplete carbonization, which increased with lower PYT (Table S1†). Thus, it would be possible for the incomplete carbonization to undergo further carbonization *via* a 2nd pyrolysis during activation. Therefore, an attempt was made to demonstrate the 2nd pyrolysis *via* the heat treatment (HT) of the CMs under same thermal conditions as those for the activation, with the exception of KOH impregnation. The results showed a weight loss of 15.4, 6.8, 4.1 and 2.3% for HC-45-500, HC-45-600, HC-45-700 and HC-45-800, respectively. It is believed that the weight loss occurred due to the 2nd pyrolysis of the incomplete carbonization even in the absence of KOH. This is supported by the fact that the losses increased with lower PYT, similar to the trend of increased incomplete carbonization with lower PYT. These weight losses are smaller than those from the activation due to the absence of KOH. It was observed that ∼5% difference in the weight loss existed between activation and HT, regardless of the PYT. This can be related to the similar KOH gains (94%), irrespective of PYT (Table S3†).

TGA analysis was performed to provide a better understanding of the pyrolysis, heat treatment and activation processes. A very small weight loss of ∼4% was observed for CM-45-700 and CM-45-800 up to 700 °C (Fig. S1a†). On the other hand, CM-45-500 and CM-45-600 showed a very small weight loss up to 450 or 500 °C, followed by a rapid or relatively rapid loss, exhibiting 16.1 and 7.4% at 700 °C, respectively. Such trends are similar to those reported by Kim and co-workers.^28^ These losses are similar to the ones observed from the HT process (15.4, 6.8, 4.1 and 2.3%), and thus, the losses can be attributed to the 2nd pyrolysis upon the TGA analysis. The 2nd scan TGA of these samples, however, showed no major weight loss up to 500 °C and then exhibited a slow loss thereafter, providing 6.8, 5.2, 4.1 and 3.1% at 700 °C for CM-45-500, CM-45-600, CM-45-700 and CM-45-800, respectively (Fig. S1b†). These are very similar to their 3rd scan TGA, indicating that complete carbonization occurred *via* the 2nd TGA. The weight loss in the 2nd and 3rd TGA can be attributed to the thermal degradation of oxygen functional moieties, as observed from the graphene oxide.^29^

The HCs exhibited similar 1st and 2nd TGA scans (Fig. S1c†), unlike the CMs which showed different 1st and 2nd scans. This discrepancy can be explained by the 2nd pyrolysis that already occurred during the HT process. Thus, no 2nd pyrolysis was observed in the 1st scan TGA of HC. The ACs exhibited a similar weight loss behaviour as the HCs in the TGA study, with the exception of a small shoulder at ∼200 °C and slightly faster weight loss thereafter (Fig. S1d†). The shoulder can be attributed to the loss of the functional moieties which were introduced by activation,^30^ while the faster loss can be explained by the decomposition of stable oxygen functional groups.^31^ This resulted in the weight loss of 10.6, 9.1, 8.6 and 8.2% at 700 °C for AC-45-500, AC-45-600, AC-45-700 and AC-45-800, respectively. These weight losses are higher than the ones observed from the HCs, showing a difference of ∼4% regardless of the PYT. This can be compared with the weight loss difference of ∼5% between activation and heat treatment, as discussed above. As expected, the 2nd scan TGA of the ACs is similar to the 2nd scan TGA of the CMs, as well as the 1st scan TGA of the HCs, which is again attributed to the loss of functional groups in the 1st TGA scan of the ACs. When the R/W ratio was varied, similar TGA scans were obtained (not included here), which was expected given the similar weight loss observed upon pyrolysis (Table S1†).

**Fig. 1:**
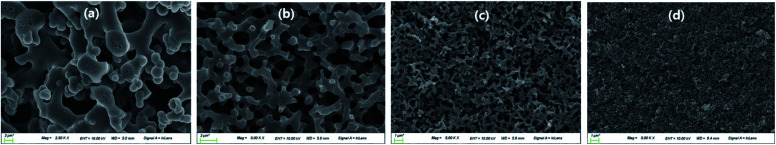
SEM micrographs of the AC-45-500 (a), AC-50-500 (b), AC-55-500 (c) and AC-60-500 (d).

In the FE-SEM analysis, the ACs exhibited an interconnected pore structure. Their pore size and interconnectivity decreased with higher R/W ratios (Fig. 1), but no change occurred with the variation of PYT (Fig S2†). For example, AC-45-500 exhibited good interconnectivity with large pores (∼3 μm in diameter, named “super macro-pores”), as shown in Fig. 1a. In comparison, almost discrete pores of 0.2–0.3 μm in diameter were obtained from AC-60-500 (Fig. 1d). This trend can be explained by the increased viscosity of the reactants with higher R/W ratio, resulting in incomplete spinodal decomposition.^32^ The decreased pore size and interconnectivity with higher R/W ratio can be related to the decreased KOH impregnation, as discussed above (Table S3†). On the other hand, similar pore size and interconnectivity, regardless of the PYT (Fig. S2†), can be attributed to the same R/F ratio and thus similar spinodal decomposition behaviour, as reported previously.^24^ This may explain the similar KOH impregnation for all PYT, as discussed above (Table S3†).

**Fig. 2:**
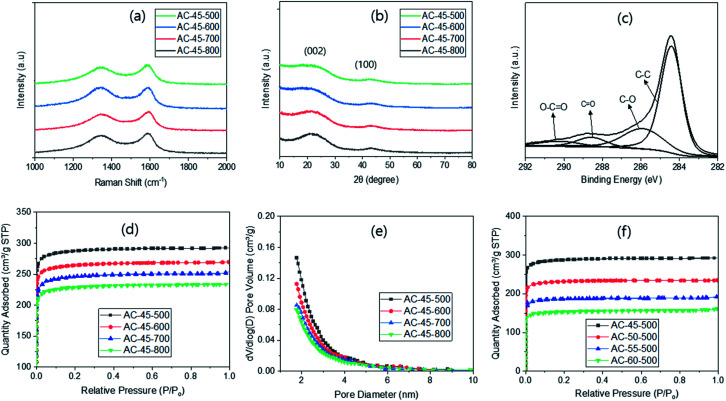
Characterization of ACs *via* Raman (a), XRD (b), XPS (c), N_2_ sorption measurement with R/W variation (d) and pore size distribution (e), and N_2_ sorption measurement with PYT variation (f).

The Raman spectroscopy of the ACs exhibited two characteristic peaks at ∼1340 and ∼1590 cm^−1^ (Fig. 2a). The former corresponds to the D-bands from the breathing mode of sp2 carbons on the edges or defects and the latter to the G-bands from the stretching motion of sp^2^ carbons.^33^ Since the intensity ratios of these peaks (*I*_D_/*I*_G_) are known to be related to the degree of disorder, they were calculated, yielding 2.22, 1.88, 1.75 and 1.64 for AC-45-500, AC-45-600, AC-45-700 and AC-45-800, respectively. The *I*_D_/*I*_G_ ratio decreased with higher PYT, indicating higher ordering with higher PYT. Few studies also reported a decreased trend^34,35^ but the general trend in the literature is increased *I*_D_/*I*_G_ ratios upon activation.^35^ It is interesting to note that the *I*_D_/*I*_G_ ratios from the ACs are very similar to the ratios from the corresponding CMs, which exhibited 2.17, 1.85, 1.74 and 1.66 for CM-45-500, CM-45-600, CM-45-700 and CM-45-800, respectively (Fig. S4a†).

Such unusual trend observed in this study can be explained by (1) no true increase in the *I*_D_/*I*_G_ ratio upon activation, or (2) nullification of the increased *I*_D_/*I*_G_ ratio upon activation by the decreased *I*_D_/*I*_G_ ratio *via* the 2nd pyrolysis. The first hypothesis is less than likely since activation with only 0.5% KOH increased the SSA despite the lack of Raman data.^27^ On the other hand, the second hypothesis would be valid if there was a decrease in the *I*_D_/*I*_G_ ratio through some process during activation, and if this decrease was the same as the increase resulting from activation. Concerning the decrease, a clue was found in Fig. 2a, which revealed that the *I*_D_/*I*_G_ ratio decreased with higher PYT. In this regard, the 2nd pyrolysis can possibly decrease the *I*_D_/*I*_G_ ratio since it increases the degree of pyrolysis.

To confirm this, a Raman study was carried out on the HCs (Fig. S3b†). The results indicated that the samples experienced the 2nd pyrolysis upon HT, providing *I*_D_/*I*_G_ ratios of 1.65, 1.57, 1.51 and 1.45 for HC-45-500, HC-45-600, HC-45-700 and HC-45-800, respectively. As expected, these numbers are smaller than the values obtained from CMs, as a result of the 2nd pyrolysis upon HT. This demonstrates that activation not only increases the *I*_D_/*I*_G_ ratio through true activation, but also decreases it by the 2nd pyrolysis taking place during activation. On the other hand, no change in the *I*_D_/*I*_G_ ratio was observed in this study since a small increase in the *I*_D_/*I*_G_ ratio arising from the low loading of KOH (<100%) is countered by a small decrease from the 2nd pyrolysis. In this respect, a large increase in the *I*_D_/*I*_G_ ratio in the literature can be attributed to the high loading of KOH.^36^ Furthermore, a study on the effect of the R/W ratio showed similar *I*_D_/*I*_G_ ratios of 1.72, 1.73, 1.71 and 1.70 for AC-45-700, AC-50-700, AC-55-700 and AC-60-700, respectively (Fig. S3c†). This arises from the same R/F ratio and the same PYT used for all samples.

The XRD analysis on the ACs showed a strong peak at ∼22° and a weaker peak at ∼43° (Fig. 2b), respectively corresponding to (002) and (100) of the disordered graphite.^37^ As the PYT increased, the (002) peak shifted slightly to a higher diffraction angle, indicating higher ordering.^38^ This corresponds well to the results from the Raman analysis (Fig. 2a). However, there was no peak shift with the variation of R/W ratio (Fig. S4†), which relates to no change in the *I*_D_/*I*_G_ ratio in the Raman study (Fig. S4c†). This is again attributed to the same R/F ratio and the same PYT used. It is also interesting to note that no appreciable decrease in the intensity of the (002) peak was observed upon activation in this study. This can be compared with almost complete disappearance of the peak upon activation reported in the literature.^39^ This discrepancy may arise from the low loading of KOH (<100%) in this study, which in turn means a low degree of activation, compared with 400% KOH loading in the literature.^39^

In the wide scan XPS, the ACs showed carbon (∼285 eV) and oxygen peaks (∼532 eV) (Fig. 2c), as expected from the RF-based carbon.^35^ The carbon concentrations calculated from these were 85.3, 88.9, 89.7 and 90.5% for AC-45-500, AC-45-600, AC-45-700 and AC-45-800, respectively. The increased concentration with higher PYT indicates a higher degree of pyrolysis with higher PYT (Table S1†), as reported previously.^38^ This can also be related to the decreased incomplete carbonization with higher PYT, as well as to the higher weight loss with lower PYT in the TGA analysis (Fig. S1a†). These carbon concentrations are slightly lower than those from the corresponding CMs (88.5, 92.3, 94.7 and 95.5% for CM-45-500, CM-45-600, CM-45-700 and CM-45-800, respectively), as a result of the increased oxygen functional moieties upon KOH activation. The deconvolution of C 1s peaks from the ACs provided a strong C–C peak at 284.4 eV, as well as weak C–O, C=O and O–C=O peaks at 285.9, 288.7 and 290.3 eV, respectively (Fig. 2c). The results were similar to the ones obtained from activated carbon in the literature.^35,38^ It is noted that the intensity of the three weaker peaks decreased slightly with higher PYT (Fig. S5†), indicating decreased oxygen functional moieties with higher PYT.^35,38,40^

The N_2_ sorption of the ACs was also studied as a function of PYT (Fig. 2d). One can see Type I isotherms for all PYT, suggesting the presence of micro- and meso-pores, which is also supported by the pore size distribution (Fig. 2e). This can be compared with Type IV isotherms observed from the KOH activated carbon materials in the literature,^41^ which indicate the presence of not only micro and meso-pores but also macro-pores, possibly due to the high loading KOH. In this respect, the Type I isotherm obtained in this study can be attributed to the low loading of KOH (<100%). The specific surface areas (SSAs) calculated based on these adsorption data were 1173, 1081, 997 and 929 m^2^ g^−1^ for AC-45-500, AC-45-600, AC-45-700 and AC-45-800, respectively (Table 1). The SSA increased with lower PYT, showing a maximum at 500 °C pyrolysis. This can be attributed to the increased pore generation with lower PYT *via* the 2nd pyrolysis. This is also supported by the increased weight loss observed with lower PYT upon activation, even though the KOH gain remained the same regardless of the PYT (Table S3†). This is similar to the highest SSA reported in one study, which was obtained from 400 °C pyrolysis and activation at 800 °C after 100% KOH soaking.^42^

**Table 1:** Characteristics of ACs with pyrolysis temperature variation at R/W = 45

	*S* a (m^2^ g^−1^)	*V*_total_ b (cm^3^ g^−1^)	*V*_micro_ c (cm^3^ g^−1^)	APD^d^ (nm)
AC-45-500	1173	0.454	0.451 (99.3%)	2.51
AC-45-600	1081	0.418	0.414 (99.0%)	2.56
AC-45-700	997	0.389	0.383 (98.5%)	2.73
AC-45-800	929	0.363	0.357 (98.4%)	2.97

a Specific surface area.

b Total pore volume.

c Micro pore volume.

d Average pore diameter.

However, most studies in the literature reported a maximum SSA athighertemperaturessuchas700,^43^ 800^44,45^ or 900 °C.^46,47^ The discrepancy of our data may be explained by the 2-step process (pyrolysis and then activation) employed in this study as opposed to the 1-step process (simultaneous pyrolysis and activation) used in the literature. In the 1-step process, higher SSA results from the higher weight loss at higher temperatures, as observed from the pyrolysis in this study (Table S1†). On the other hand, in the 2-step process, the increased weight loss is observed with higher temperatures in the first pyrolysis stage, just as in the 1-step process. In the second activation stage (the 2nd step), however, the increased weight loss is observed with lower PYT, because of the 2nd pyrolysis of the incomplete carbonization. This leads to increased pore generation with lower PYT, and thus, higher SSA.

For additional confirmation, the CMs were subjected to a N_2_ sorption study (Fig. S7a†). As expected, the SSA increased with PYT (556, 576, 598 and 601 m^2^ g^−1^ for CM-45-500, CM-45-600, CM-45-700 and CM-45-800, respectively (Table S5†)), as a result of better pore generation at higher PYT.^35^ These values are also similar to those reported in the literature for RF-based carbon with no activation.^38^ However, these SSAs are much smaller than the values obtained from the ACs, demonstrating the effect of KOH on the activation. The N_2_ sorption of the ACs was also measured as a function of the R/W ratio. The results showed that the N_2_ sorption decreased with the R/W ratio (Fig. 2f), being attributed to lower KOH impregnation with higher R/W, and thus, a lower degree of activation, as evidenced by the lower weight loss (Table S4†). The SSAs calculated based on these isotherms were 1173, 938, 742 and 619 m^2^ g^−1^ for AC-45-500, AC-50-500, AC-55-500 and AC-60-500, respectively (Table S6†).

**Fig. 3:**
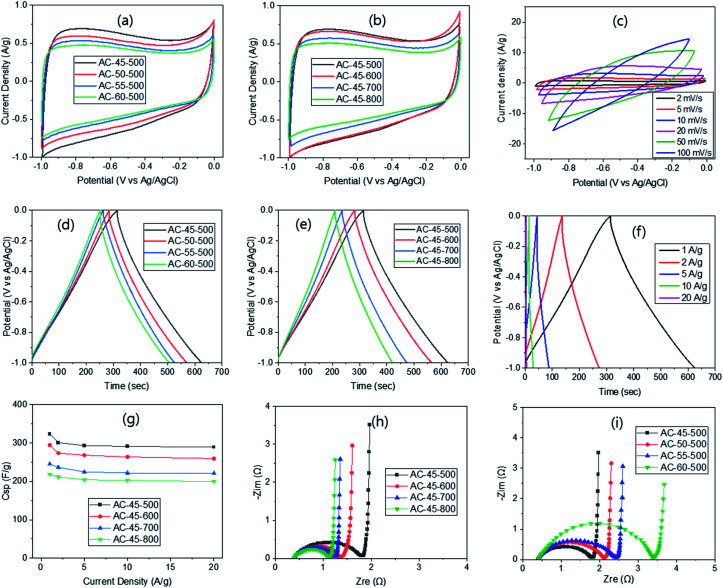
Electrochemical properties of ACs *via* the 3-electrode system; CV with R/W (a), PYT (b) and scan rate variation for AC-45-500 (c), GCD with R/W (d), PYT (e) and current density variation for AC-45-500 (f), capacitance change with current density variation (g), and Nyquist plots with PYT (h) and R/W variation (i).

The electrochemical properties were evaluated in 6 M KOH solution *via* the three-electrode system first. Cyclic voltammetry (CV) carried out at 2 mV s^−1^ exhibited rectangular shape curves, irrespective of the R/W ratio (Fig. 3a), displaying typical voltammograms of electrical double-layer capacitors. But the size of the curve increased slightly as the R/W ratio decreased with the largest one at R/W = 45, as expected from the highest SSA at R/W = 45 (Table S6†). A small hump observed on the CV curves can be attributed to the pseudo-faradaic reaction arising from the oxygen functional moieties on the electrode surface,^43^ as supported by the XPS analysis (Fig. 2c). When the PYT was varied, the rectangular shape CV curve was still observed, but its size increased as the PYT decreased. The largest one was observed at 500 °C (Fig. 3b), as expected from its highest SSA (Table 1). This can be compared with the largest CV curve from 700 (ref. 43) or 800 °C (ref. 44) reported in the literature. The difference is again attributed to the 2-step process used in this study, compared with the 1-step process in the literature, as discussed above. Additionally, the scan rate variation was also attempted from 2 to 100 mV s^−1^ with AC-45-500, which had the largest CV curve. The resulting CV curve showed a quasi-rectangular shape (Fig. 3c), as also reported elsewhere.^45^

The GCD test was also performed at 1 A g^−1^ current density and displayed isosceles triangular shape curves (Fig. 3d), which are characteristics of the electric double-layer capacitors.^48^ In addition, a sharp response with a small IR drop was observed, indicating an ideal capacitive behaviour and a relatively small internal resistance, respectively. As noted, the discharge time and the IR drop decreased with decreasing R/W ratio, exhibiting the smallest value at R/W = 45, as expected from the largest CV curve. This can be related to the interconnected pore structure with super-macro pores, which promoted the easy flow of electrolyte. Based on these GCD curves, the specific capacitance (*C*_sp_) was calculated to be 261, 247, 215 and 211 F g^−1^ for AC-45-700, AC-50-700, AC-55-700 and AC-60-700, respectively. These values correspond well to the higher SSAs with lower R/W ratios (Table S6†). On the other hand, when the PYT was varied at R/W = 45, the discharge time decreased and the IR drop increased with increasing PYT (Fig. 3e), being related to decreased SSA with increased PYT. Nevertheless, the GCD curves still maintained their isosceles triangular shape, keeping the characteristic of the electric double-layer capacitor. The calculated *C*_sp_ was 218, 247, 295 and 323 for AC-45-800, AC-45-700, AC-45-600 and AC-45-500, respectively, and increased with decreasing PYT. For the purpose of comparison, the values reported are 294 (ref. 49) and 324 F g^−1^ (ref. 50) for the RF-based ACs. It is interesting to note that the latter study exhibited a similar *C*_sp_ value despite its much higher SSA of 3418 m^2^ g^−1^ (1173 m^2^ g^−1^ in this study). It suggests that the micro-pores in the latter study are too small to be accessed by the electrolyte but large enough to be filled with N_2_ molecules. In contrast, the samples in this study, which possess an interconnected pore structure with super-macro pores (∼3 μm) (Fig. 1), provide an easy flow of the electrolyte, leading to high capacitance values despite the much lower SSA. The current density variation in the range of 1–20 A g^−1^ for AC-45-500 produced isosceles triangular shape GCD curves with decreased discharge time (Fig. 3f). This can be attributed to the hindered flow of electrolyte at high current density, and thus, increased internal resistance.^48^ The *C*_sp_ calculated from these curves were 323, 301, 293, 291 and 289 F g^−1^ for 1, 2, 5, 10 and 20 A g^−1^, respectively. This resulted in 89.5% retention at 20 A g^−1^ (Fig. 3g). As the PYT increased, similar retentions were observed, exhibiting 88.1, 90.1 and 91.3% for AC-45-600, AC-45-700 and AC-45-800, respectively (Fig. 3g), despite the decreased capacitance. Excellent retention obtained in this study can again be attributed to the interconnected super-macro pores (∼3 μm), as discussed above.

In addition, the electrochemical impedance spectroscopy (EIS) was carried out on the ACs in the frequency range of 0.01–100 kHz with the aqueous 6 M KOH solution in order to understand the diffusion kinetics of the electrolyte. The corresponding Nyquist plots (Fig. 3h) exhibited a small semicircle in the high frequency range, Warburg diffusion in the mid-frequency range and a vertical line in the low frequency range. In the high-frequency region, the first interception points were observed at ∼0.4 Ω, irrespective of the PYT. This is known to be equivalent serial internal resistance (*R*_s_) which is related to the resistance of the electrolyte to the electrode.^39^ Similar *R*_s_ values can be explained by the same R/F ratio, and thus, similar property of the ACs. The radius of the semicircle in the high frequency range, known to be the charge transfer resistance (*R*_ct_), was also measured. The values obtained were 1.41, 0.94, 0.87 and 0.72 Ω for AC-45-500, AC-45-600, AC-45-700 and AC-45-800, respectively (Fig. 3h). The decreased R_ct_ can be related to the smaller IR drop with higher PYT from the GCD study (Fig. 3e), as well as to the smaller *I*_D_/*I*_G_ ratio in the Raman study. The very small Warburg impedance, irrespective of the PYT, can be attributed to the low diffusive resistance of the electrolyte, which is again related to the interconnected super-macro pores. The vertical line in the low-frequency range also arises from the interconnected super-macro pore structure, which promotes ideal capacitance behaviour.^50^ Similar Nyquist plots were obtained when the R/W ratio was varied. The increased *R*_ct_ with higher R/W ratio (Fig. 3i) can be related to the increased IR drop with higher R/W ratio (Fig. 3d), as well as to the decreased pore size and interconnectivity (Fig. 1) and the decreased SSA with higher R/W ratio (Table S5†).

**Fig. 4:**
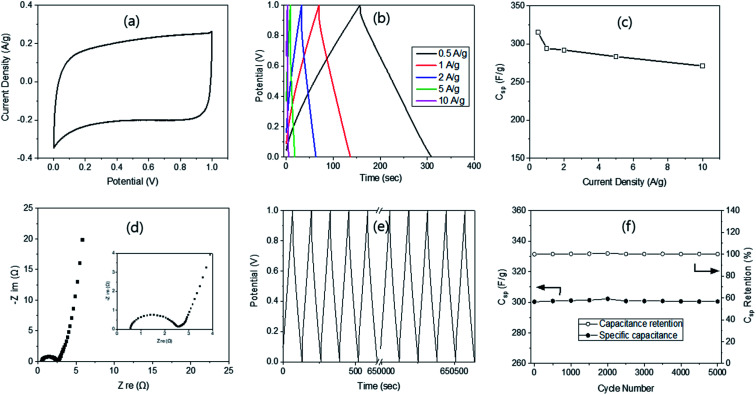
Electrochemical properties of ACs (R/W = 45) *via* the 2-electrode systems; CV (a), GCD (b), capacitance with current density change (c), Nyquist plots (d), cyclic study (e) and capacitance and its retention *via* the cyclic test (f).

Finally, a two-electrode cell was also fabricated, but only with ACM-45-500 since it has the highest *C*_sp_. The CV curve obtained displayed a typical rectangular shape (Fig. 4a), just as in the three-electrode system, indicating the formation of electrical double layer. However, it was approximately half in size as that from the 3-electrode system (Fig. 3a) because of the doubled weight of the two electrodes compared with the single electrode weight used in the 3-electrode system. The curve became a quasi-rectangular shape with higher scan rate (Fig. S8†), still showing the electrical double layer formation. The GCD curves were also obtained at the current density ranging from 1 to 10 A g^−1^ (20 A g^−1^ was not possible due to the doubled weight of the electrode). The curves were symmetrical in shape and possessed a nearly linear discharge slope (Fig. 4b). These are again similar to the ones obtained from the three-electrode system, indicating excellent electrochemical double-layer behaviour. The specific capacitance was calculated based on the GCD curves, as reported previously,^26^ providing 303 F g^−1^ at 1 A g^−1^, which is slightly lower than 323 F g^−1^ at 1 A g^−1^ from the three-electrode system. The *C*_sp_ decreased with increasing current density, going from 287 to 281 and 271 F g^−1^ at 2, 5 and 10 A g^−1^, respectively. The retention was 89.4% at 10 A g^−1^ (Fig. 4c), compared with 90.1% from the 3-electrode system (1–10 A g^−1^).

The Nyquist plot from the EIS provided a semi-circle in the high frequency region, very small Warburg impedance in the mid-frequency region and a nearly vertical line in the low-frequency region (Fig. 4d), demonstrating the ideal capacitive behaviour of electrochemical double-layer capacitors. Moreover, a very small *R*_s_ value of 0.5 Ω was measured, indicating good electron conduction and fast ion exchange between the electrode and the electrolyte. The *R*_ct_ was found to be 2.13 Ω, which is much larger than 1.41 Ω from the 3-electrode system, possibly due to the two electrodes. The cycling stability of AC-45-500 was evaluated at 1 A g^−1^ (Fig. 4e) and showed 100% remain in capacitance even after 5000 cycles of the charging–discharging process (Fig. 4f). Such high remain can be compared with 100 (10k cycle),^47^ 99.5 (10k cycle),^46^ 96.5 (3k cycle),^43^ 94.6 (10k cycle),^40^ and 87.2% (20k cycle)^48^ reported in the literature. The high retention in this study can be explained by the interconnected pore structure with super-macro pores, as explained above. The energy and power densities of the supercapacitor with AC-45-500 were calculated according to the method reported in the literature^25^ and resulted in a maximum energy density of 8.7 W h kg^−1^ at the power density of 467.5 W kg^−1^. These values can be compared with 10.5 W h kg^−1^ @ 1.4 kW kg^−1^ from RF by Z. B. Wen.^51^ The results demonstrate that AC-45-500 is a promising electrode material for electrochemical supercapacitors.

## Conclusions

Activated carbon xerogel monoliths (ACs) were successfully prepared from resorcinol and formaldehyde *via* the catalyst-free and template-free hydrothermal polycondensation reaction, followed by pyrolysis at 500, 600, 700 or 800 °C for 4 h and then activation at 700 °C for 1 h after impregnation in the KOH solution. The CV study of the ACs displayed a typical voltammogram of the electrical double-layer capacitors, while the GCD study showed isosceles triangular shape curves, which are characteristics of the electric double-layer capacitors. Based on the GCD study, a high specific capacitance (*C*_sp_) of 323 F g^−1^ was obtained at R/W = 45 and PYT of 500 °C. The specific capacitance decreased with higher PYT, as well as higher R/W ratio. As noted, this is an opposite trend to those in the literature and can be explained by the 2nd pyrolysis from the 2-step process (pyrolysis and then activation) in this study, which provided extra pores. The *C*_sp_ decreased with higher R/W due to the decreased pore size and interconnectivity, which resulted in a higher flow resistance of the electrolyte. AC-45-500 exhibited 89.4% retention at 20 A g^−1^, which is attributed to the interconnected super macro pore structure. The Nyquist plot from the EIS study exhibited a small semicircle in the high frequency range, steep Warburg diffusion in the mid-frequency range and a vertical line in the low frequency range. These results can again be attributed to the interconnected super-macro pores. The RS2032 coin cells from AC-45-500 exhibited very similar results as those obtained with the 3-electrode system, and 100% retention in capacitance was observed after 5000 cycling test at the current density of 1 A g^−1^. The energy and power density calculated based on the coin cell study was 8.7 W h kg^−1^ and 467.5 W kg^−1^, respectively, demonstrating the excellent electrochemical properties of the ACs.

## Conflicts of interest

There are no conflicts to declare.

## Supplementary Material

RA-011-D1RA06462B-s001

## References

[cit1] Shao H., Wu Y.-C., Lin Z., Taberna P.-L., Simon P. (2020). Chem. Soc. Rev..

[cit2] Najib S., Erdem E. (2019). Nanoscale Adv..

[cit3] Sharma P., Kumar V. (2020). J. Electron. Mater..

[cit4] Yang X. Y., Chen L.-H., Li Y., Rooke J. C., Sanchezc C., Su B.-L. (2017). Chem. Soc. Rev..

[cit5] Gonzalez A., Goikolea E., Barrena J. A., Mysyk R. (2016). Renewable Sustainable Energy Rev..

[cit6] Choi C., Ashby D. S., Butts D. M., DeBlock R. H., Wei Q., Lau J., Dunn B. (2020). Nat. Rev. Mater..

[cit7] Veerakumar P., Sangili A., Manavalan S., Thanasekaran P., Lin K.-C. (2020). Ind. Eng. Chem. Res..

[cit8] Bi Z., Kong Q., Cao Y., Sun G., Su F., Wei X., Li X., Ahmad A., Xie L., Chen C.-M. (2019). J. Mater. Chem. A.

[cit9] Wang Y., Qu Q., Gao S., Tang G., Liu K., He S., Huang C. (2019). Carbon.

[cit10] Yang H., Ye S., Zhou J., Liang T. (2019). Front. Chem..

[cit11] Li D., Tian Y., Qiao Y., Wen L. (2014). Mater. Lett..

[cit12] Dolah B. N. M., Deraman M., Othman M. A. R., Farma R., Taer E., Awitdrus, Basri N. H., Talib I. A., Omar R., Nor N. S. M. (2014). Mater. Res. Bull..

[cit13] Yu M., Han Y., Li Y., Li J., Wang L. (2018). Carbohydr. Polym..

[cit14] Pekala R. W., Farmer J. C., Alviso C. T., Tran T. D., Mayer S. T., Miller J. M., Dunn B. (1998). J. Non-Cryst. Solids.

[cit15] Huang Y., Cai H., Feng D., Gu D., Deng Y., Tu B., Wang H., Webley P. A., Zhao D. (2008). Chem. Commun..

[cit16] Brun N., García-González C. A., Smirnova I., Titirici M. M. (2013). RSC Adv..

[cit17] Al-Muhtaseb S. A., Ritter J. A. (2003). Adv. Mater..

[cit18] Baumann T. F., Worsley M. A., Han T. Y. J., Satcher J. H. (2008). J. Non-Cryst. Solids.

[cit19] Hasegawa G., Aoki M., Kanamori K., Nakanishi K., Hanada T., Tadanaga K. (2011). J. Mater. Chem..

[cit20] Liu C., Han G., Chang Y., Xiao Y., Li M., Zhou W. (2016). Electrochim. Acta.

[cit21] Gupta K., Liu T., Kavian R., Chae H. G., Ryu G. H., Lee Z., Lee S. W., Kumar S. (2016). J. Mater. Chem. A.

[cit22] Adelhelm P., Hu Y.-S., Chuenchom L., Antonietti M., Smarsly B. M., Maier J. (2007). Adv. Mater..

[cit23] Li F., Xie L., Sun G., Kong Q., Su F., Cao Y., Wei J., Ahmad A., Guo X., Chen C.-M. (2019). Microporous Mesoporous Mater..

[cit24] Yoon H. J., Lee J. Y., Lee J. S., Yoon T. H. (2019). RSC Adv..

[cit25] Wang Y. G., Xia Y. Y. (2006). J. Electrochem. Soc..

[cit26] Stoller M. D., Rouff R. S. (2010). Energy Environ. Sci..

[cit27] Abouelamaiem D. I., Mostazo-López M. J., Hec G., Patel D., Neville T. P., Parkin I. P., Lozano-Castelló D., Morallón E., Cazorla-Amorós D., Jorge A. B., Wang R., Ji S., Titirici M.-M., Shearing P. R., Brett D. J. L. (2018). J. Energy Storage.

[cit28] Kim J. M., Song I. S., Cho D., Hong I. (2011). Carbon Lett..

[cit29] Soltani T., Lee B. K. (2017). J. Colloid Interface Sci..

[cit30] de la Puenta G., Pis J. J., Menendez J. A., Grange P. (1997). J. Anal. Appl. Pyrolysis.

[cit31] Zhang G., Wen M., Wang S., Chen J., Wang J. (2018). RSC Adv..

[cit32] Scherdel C., Scherb T., Reichenauer G. (2009). Carbon.

[cit33] Nakamizo M., Honda H., Inagaki M. (1978). Carbon.

[cit34] Serwar M., Rana U. A., Siddiqi H. M., Ud-Din Khan S., Ahmed Ali F. A., Al-Fatesh A., Adomkevicius A., Coca-Clemente J. A., Cabo-Fernandez L., Bragab F., Hardwick L. J. (2017). RSC Adv..

[cit35] Zhang Q., Deng X., Ji M., Li Y., Shi Z. (2020). Ionics.

[cit36] Yu K., Zhu H., Qi H., Liang C. (2018). Diamond Relat. Mater..

[cit37] Bhattacharjya D., Yu J. S. (2014). J. Power Sources.

[cit38] Goel C., Bhunia H., Bajpai P. K. (2015). RSC Adv..

[cit39] Cheng Y., Wu L., Fang C., Li T., Chen J., Yang M., Zhan Q. (2020). J. Mater. Res. Technol..

[cit40] Zhao H., Xing B., Zhang C., Huang G., Liu Q., Yi G., Jia J., Ma M., Chen Z., Zhang C. (2018). J. Alloys Compd..

[cit41] Zhang K., Ang B. T., Zhang L. L., Zhao X. S., Wu J. (2011). J. Mater. Chem..

[cit42] Cheng J., Xu Q., Wang X., Li Z., Wu F., Shao J., Xie H. (2019). Sustainable Energy Fuels.

[cit43] Zhang X., Shen N., Yao Z., Wu R. (2020). RSC Adv..

[cit44] Wei X., Jiang X., Wei J., Gao S. (2016). Chem. Mater..

[cit45] Zhao Z., Wang Y., Li M., Yang R. (2015). RSC Adv..

[cit46] Zhou Y., Ren J., Xia L., Zheng Q., Liao J., Long E., Xie F., Xu C., Lin D. (2018). Electrochim. Acta.

[cit47] Fasakin O., Dangbegnon J. K., Momodu D. Y., Madito M. J., Oyedotun K. O., Eleruja M. A., Manyala N. (2018). Electrochim. Acta.

[cit48] Wei T., Zhang Q., Wei X., Gao Y., Li H. (2016). Sci. Rep..

[cit49] Zhu Y., Hu H., Li W., Zhang X. (2007). Carbon.

[cit50] Chang Y. M., Wu C. Y., Wu P. W. (2013). J. Power Sources.

[cit51] Wen Z. B., Qu Q. T., Gao Q., Zheng X. W., Hu Z. H., Wu Y. P., Liu Y. F., Wang X. K. (2009). Electrochem. Commun..

