# Supplementary material for: Electrochemical properties of an activated carbon xerogel monolith from resorcinol–formaldehyde for supercapacitor electrode applications
Source: RSC Adv. 2021 Oct 8;11(53):33192–201. doi: 10.1039/d1ra06462b (PMC9042302; doi:10.1039/d1ra06462b)
Supplement: RA-011-D1RA06462B-s001 [file RA-011-D1RA06462B-s001.pdf]

Electronic Supplementary Material (ESI) for RSC Advances.
This journal is © The Royal Society of Chemistry 2021

Electronic Supplementary Information (ESI)

Electrochemical property of activated carbon xerogel monolith from resorcinol and formaldehyde for supercapacitor electrode applications

Minhu Huang, Seung Joon Yoo, Jae-Suk Lee and Tae-Ho Yoon*

School of Materials Science and Engineering, Gwangju Institute of Science and Engineering, Gwangju, 61005, South Korea.

* To whom correspondence should be addressed (thyoon@gist.ac.kr, Fax: +82-62-715-2304, Tel:+82-62-715-2307)

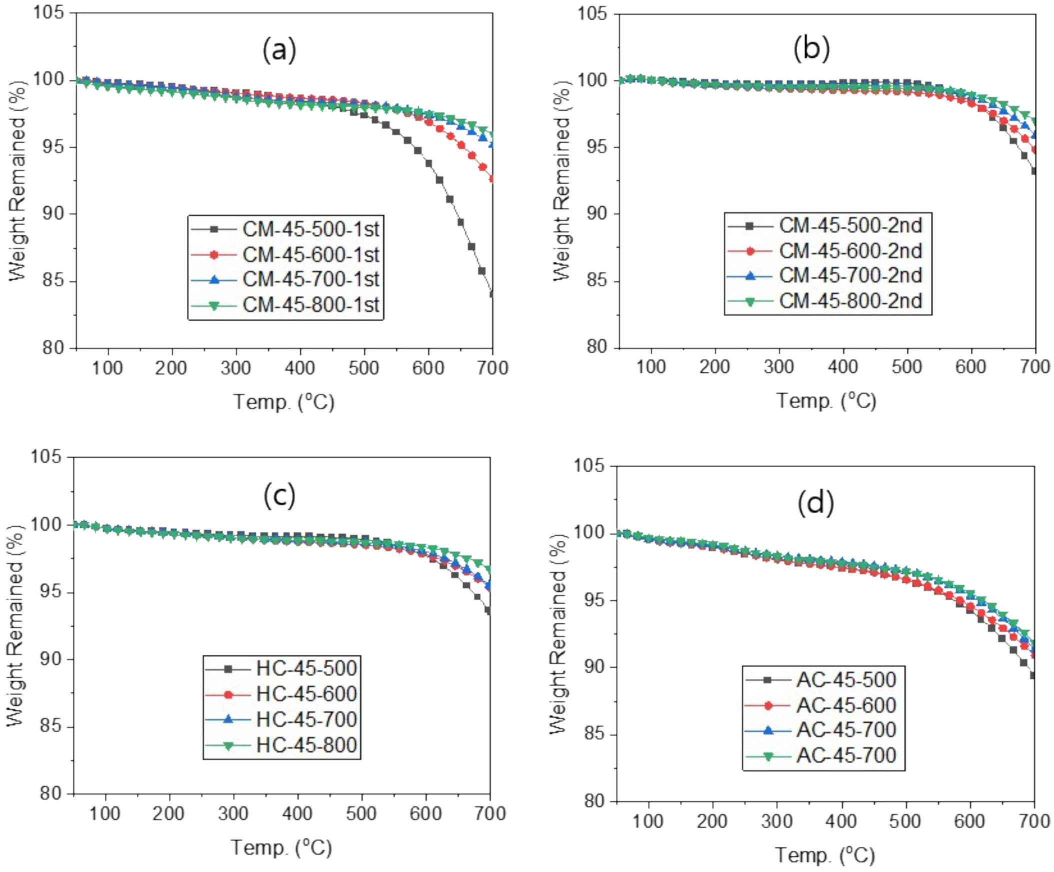

Fig. S1. 1st TGA scan (a) and 2nd TGA scan (b) of CM, and 1st TGA scans of HT (c) and AC (d) at 10 °C/min under N_2 flow

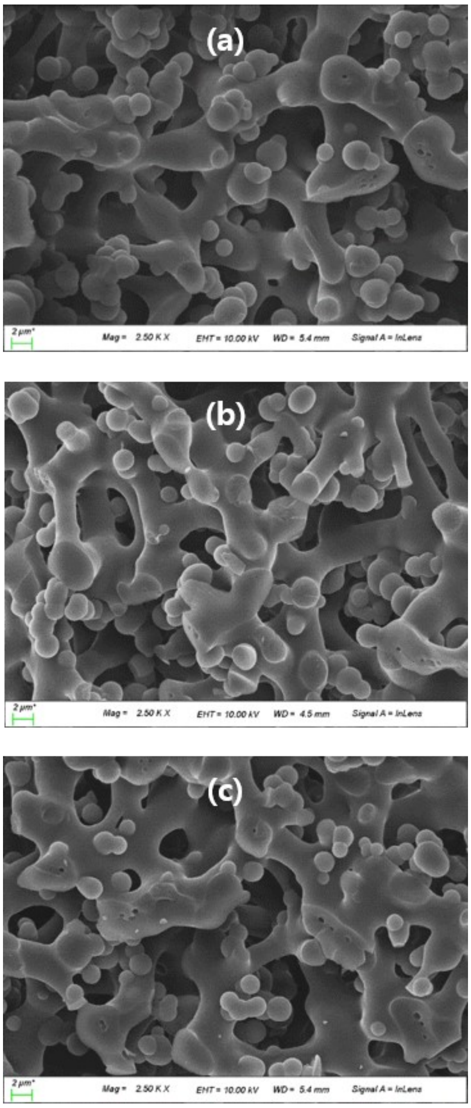

Fig. S2. SEM micrographs of AC after pyrolysis at 600 (a), 700 (b), and 800 °C (c) at R/W=45

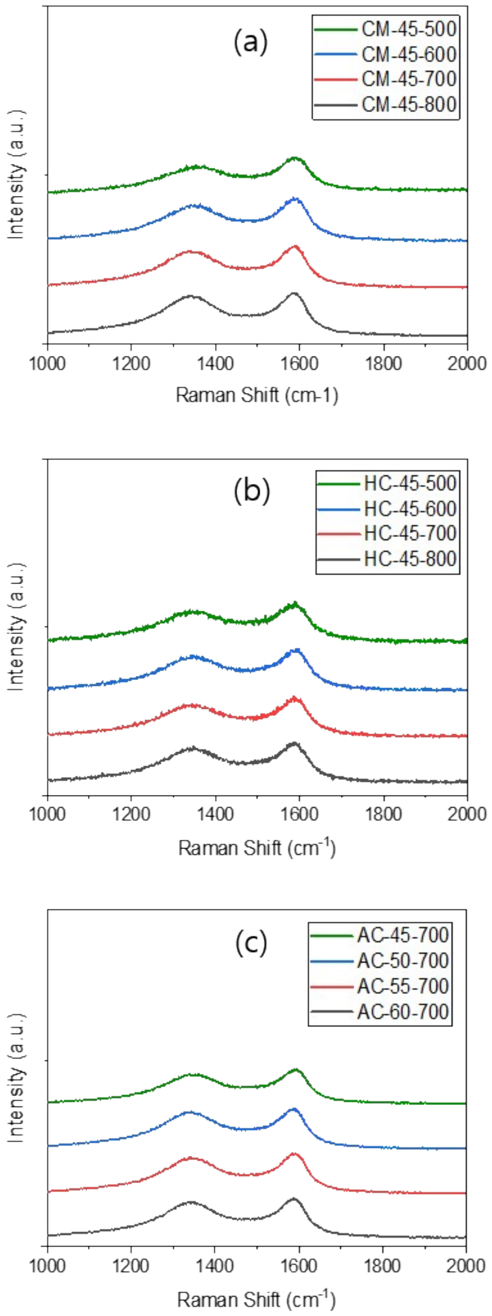

Fig. S3. Raman spectroscopy of CM (a) and HT (b) as a function of PYT at R/W=45, and AC (c) as a function of R/W ratio at 700 °C pyrolysis

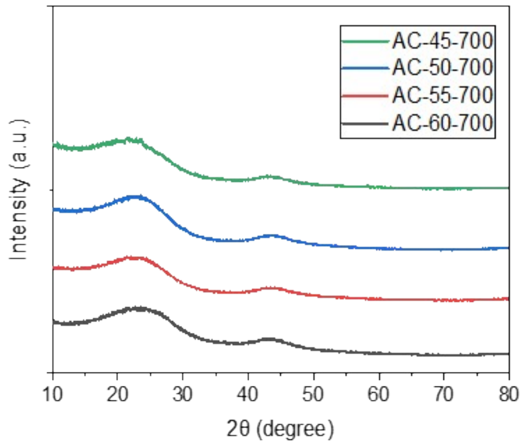

Fig. S4. XRD of AC as a function of R/W ratio at 700 °C pyrolysis

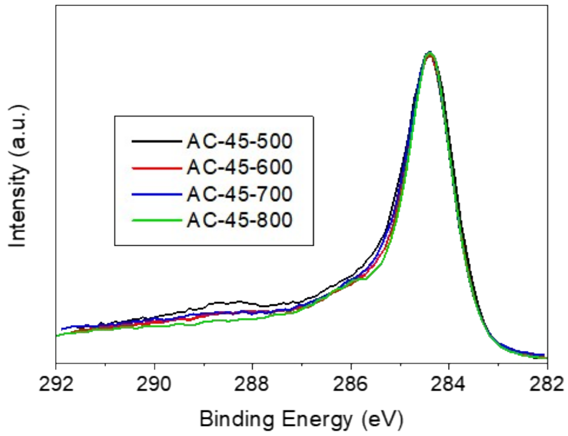

Fig. S5. C_{1s} peaks of AC as a function of PYT at R/W=45

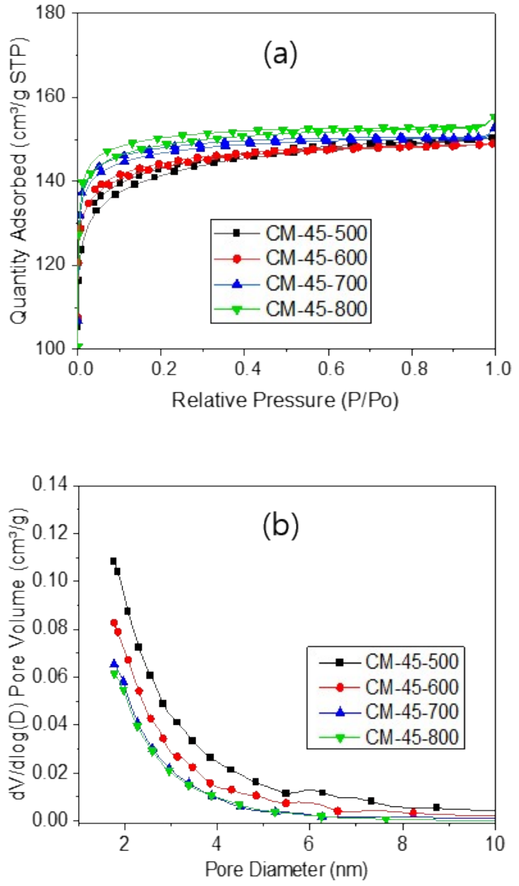

Fig. S7. N_2 sorption (a) and pore size distribution (b) of CM as a function of PYT at R/W=45

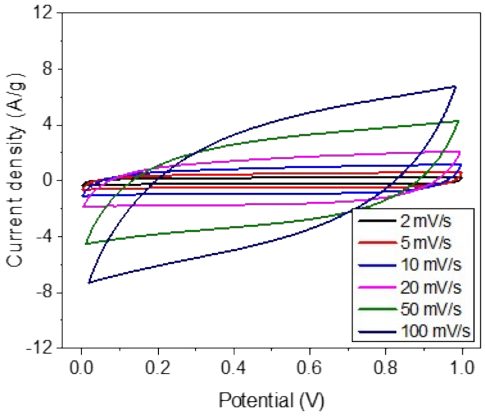

Fig. S8. Scan rate variation of AC-45-500 from the two-electrode system

Table S1. Weight loss (%) of CM upon pyrolysis

	R/W=45	R/W=50	R/W=55	R/W=60
CM-xx-500	31.7	32.5	32.9	33.2
CM-xx-600	40.5	41.6	41.8	41.5
CM-xx-700	45.6	45.8	45.8	45.3
CM-xx-800	48.4	48.1	47.7	47.6

Table S2. Diameter shrinkage (%) of CM upon pyrolysis

	R/W=45	R/W=50	R/W=55	R/W=60
CM-xx-500	9.3	9.5	10.3	10.6
CM-xx-600	13.5	14.5	15.5	15.2
CM-xx-700	16.8	17.5	17.9	17.9
CM-xx-800	18.7	19.6	19.4	19.9

Table S3. Weight gain (%) of CM upon KOH impregnation

	R/W=45	R/W=50	R/W=55	R/W=60
CM-xx-500	94.5	42.8	22.3	19.6
CM-xx-600	93.3	43.3	23.8	19.3
CM-xx-700	94.8	44.1	23.4	19.4
CM-xx-800	94.4	43.4	22..5	21.1

Table S4. Weight loss (%) of AC upon activation at 700 °C for 1 h

	R/W=45	R/W=50	R/W=55	R/W=60
AC-xx-500	20.1	17.3	13.9	11.9
AC-xx-600	10.4	6.9	4.2	2.6
AC-xx-700	8.3	5.5	2.9	1.4
AC-xx-800	6.7	3.9	2.4	1.3

Table S5. Pore characteristics of AC as a function of R/W ratio at 500 °C pyrolysis

	S^a (m^2 g^{-1})	V_{total}^b (cm^3 g^{-1})	V_{micro}^c (cm^3 g^{-1})	APD^d (nm)
CM-45-500	556	0.233	0.229 (98.3%)	1.68
CM-45-600	576	0.231	0.228 (98.7%)	1.63
CM-45-700	598	0.241	0.236 (97.9%)	1.61
CM-45-800	601	0.240	0.234 (97.5%)	1.59

[a]Specific surface area by BET (Brunauer-Emmett-Teller) method

[b]Total pore volume obtained by total single point adsorption of the pores less than 300 nm at *P/Po*=0.99.

[b]Micro-pore volume obtained by *t*-plot method

[d]Pore size distribution calculated by BJH (Barrett-Joyner-Halenda) method

Table S6. Pore characteristics of CM as a function of PYT at R/W=45

	S^a (m^2 g^{-1})	$V_{total}{}^b$ (cm^3 g^{-1})	$V_{micro}{}^c$ (cm^3 g^{-1})	APD[d] (nm)
AC-45-500	1173	0.454	0.451 (99.3 %)	2.51
AC-50-500	938	0.364	0.357 (98.1 %)	2.39
AC-55-500	742	0.293	0.286 (97.6 %)	2.24
AC-60-500	619	0.250	0.239 (95.6 %)	2.13

[a]Specific surface area by BET (Brunauer-Emmett-Teller) method

[b]Total pore volume obtained by total single point adsorption of the pores less than 300 nm at *P/Po*=0.99

[b]Micro-pore volume obtained by *t*-plot method

[d]Average pore diameter
